# Influence of Partially Debonded Interface on Elasticity of Syntactic Foam: A Numerical Study

**DOI:** 10.3390/ma10080911

**Published:** 2017-08-08

**Authors:** Yi Je Cho, Youngjeong Kang, Young Cheol Lee, Yongho Park, Wookjin Lee

**Affiliations:** 1 Department of Materials Science and Engineering, Pusan National University, Busandaehak-ro 63beon-gil 2, Busan 46241, Korea; yijecho@pusan.ac.kr (Y.J.C.); yhpark@pusan.ac.kr (Y.P.); 2 Korea Institute of Industrial Technology (KITECH), Namyangsan 1-gil 14, Yangsan 50635, Korea; yjkang@kitech.re.kr (Y.K.); yclee87@kitech.re.kr (Y.C.L.)

**Keywords:** syntactic foam, effective properties, computational materials science, interface, finite element method, micromechanics

## Abstract

The effect of interfacial bonding of glass hollow microspheres and a polymer matrix on the elastic properties of syntactic foam was investigated using representative volume element (RVE) models, including partially debonded interfaces. Finite element analysis, with models having different debonding geometries, was performed to numerically estimate the elastic behavior of the models. The models consisted of bonded and debonded regions of interfaces; the bonded region was treated as the perfectly bonded interface, while the Coulomb friction model was used to describe the debonded region with a small friction coefficient. The changes in the tensile and compressive moduli of the foams were investigated in terms of the degree of interfacial debonding and debonding geometry.

## 1. Introduction

Polymer-based syntactic foams are a class of composites that have hollow microspheres in a matrix material. These are usually made of a polymer matrix reinforced by glass hollow microspheres. The glass hollow microspheres consist of a thin-walled stiff outer glass shell and an inner gas, which are responsible for the unique properties of syntactic foam, including extremely low apparent density, good specific strength, and low thermal conductivity [1,2,3,4].

A key factor for tailoring and improving the properties of a composite material is to try different properties, by varying reinforcement stiffness, size, volume fraction, and degree of dispersion, as well as by improving the matrix and reinforcement interfacial strength [5,6,7]. In the case of syntactic foam, the ratio of inner to outer radius of the hollow microsphere has been identified as an additional important factor which determines its properties [8]. Since many microstructural parameters as listed above can affect the foam properties, developing analytical and/or numerical models in order to independently distinguish the effect of each parameter for the design and optimization of syntactic foam is important.

Modeling the elastic properties of syntactic foam has been attempted by numerous researchers, since they help determine the structural performance of a material. For instance, Lee and Westmann [9] analytically derived lower and upper bounds for the shear modulus of syntactic foams. Huang and Gibson [10] computed the elastic constants by solving the problem of a cube containing a hollow sphere for dilute reinforced syntactic foam. Nielsen and Landel [11,12] estimated the elastic modulus of syntactic foam by taking the apparent modulus of the hollow microsphere, in terms of their inner and outer radii, and using the conventional composite mechanics theory. Tagliavia et al. [13] used a semi-analytical approach to investigate the particle-to-particle interaction behavior in syntactic foams. These analytical models provide reasonable predictions for idealized, relatively simple microstructural configurations of the foam. For complicated microstructural model configurations, including loading conditions and materials properties, numerical methods, such as the finite element method (FEM), have been used. For example, Sanders and Gibson [14,15] used FEM models to investigate the effect of hollow sphere arrangement on elastic modulus and yield strength of the syntactic foam. Marur [16] investigated the effect of random distribution of the microspheres on the elasticity of the foam, by comparing the numerical results from a single-sphere embedded unit cell with one containing multiple spheres having random distribution. Yu et al. [17] studied the effect of microsphere clustering on elastic and yielding behavior of the syntactic foam. Bardella et al. [18] recently developed a periodic representative volume element (RVE) model including 50 microspheres to investigate the elastic properties of syntactic foam by FEM. More recently, the model has been extended to be able to capture the compressive failure behavior and dynamic response of the foam, by adopting brittle fracture criteria for the microsphere in their model [19,20,21].

It is well known that the adhesion between microspheres and a matrix affects the mechanical properties of syntactic foams. If a strong adhesive force at the interface between the microsphere and the matrix exists, an applied load on the foam could be completely transferred to the microspheres. In the FEM modeling in this case, the interface can be treated as a perfect bond in which the interfacial region is considered to have infinite stiffness and complete load transfer taking place between the matrix and the microsphere. However, the assumption of perfect bonding at the interface leads to erroneous results if the adhesion force between the microsphere and the matrix is weak. In this case, failure of the interface or debonding may occur during the fabrication or when a small load is applied, causing overestimation of the overall modulus and strength of the syntactic foam by the perfect bonding assumption of the model.

Only a few modeling studies dealing with the role of a weak interface on the properties of syntactic foams exist in the literature. Marur [22] and Yu et al. [23] used three-phase FEM models to account for the weak interface effect by introducing a thin soft interfacial phase between the microsphere and the matrix. They performed parametric studies on the elastic responses of their models by varying the thickness and stiffness of the interfacial phases in order to have good fits between the model and the experiments. The main limitation of this approach is that the thickness and the stiffness of the interfacial phase are indeed not related to any measurable microstructural feature. The interfacial phase properties had been set arbitrarily without convincing microstructural evidence, and therefore, their physical meaning is still ambiguous. Tagliavia et al. [3,24] investigated the effect of microsphere-matrix debonding on the stress state and flexural strength of the syntactic foam, using a pair of spherical-cap cracks on the top and bottom of the interface along the tensile direction. With the FEM calculations, they could analyze the effect of partial debonding, in terms of debonding angle, in detail. Tagliavia et al. [25] also systematically investigated the debonding energetics using the same concept. Shams and Porfiri [26] analytically investigated the effect of interfacial debonding on energy storage, displacement fields, and interfacial opening displacements during the deformation of syntactic foams. The above models are based on the realistic microstructural assumptions and are useful for understanding the deformation and fracture behavior of syntactic foam. However, the models are still limited to simple tensile loading cases with one possible type of interfacial debonding. For instance, the experimentally obtained compressive modulus is often much less than the theoretical value [17,18,19,20], which has not yet been studied through the numerical models with a debonded interface in the literature.

Both the compressive and tensile moduli of a partially debonded syntactic foam are probably weaker than those of a perfectly bonded syntactic foam. The extent of the reduction in the modulus may depend on the debonding geometry. This study aimed at understanding the general relationships between the debonding geometry and the overall apparent modulus of the partially debonded syntactic foam. Idealized models for three different partial debonding concepts were made and used for a parametric numerical study on the elastic properties of the syntactic foam, in terms of debonding fraction and geometry.

## 2. Geometric and Physical Model Concepts

**Figure 1:**
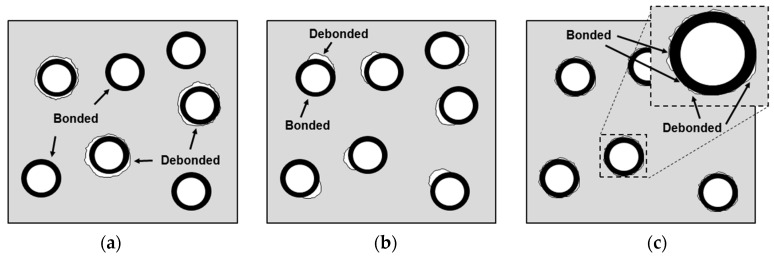
Schematics of debonding geometries for (**a**) the mixed model, (**b**) the partially debonded model and (**c**) the discontinuously bonded model.

In this study, the effect of the debonding geometry on the interface between the microsphere and the matrix was investigated by defining two interface states, bonded and debonded interfaces, in the numerical models. The bonded interface assumed a perfectly bonded state. Whereas for the debonded interface, an allowance was given for slipping and the formation of a gap between the microsphere and the matrix, thus the load transfer occurred only by contact and there was no load-transfer capacity along the debonding direction. As shown schematically in Figure 1, three types of debonding geometries were considered:

1. Mixed model (Figure 1a): There is a mixture of microspheres having both bonded and debonded interfaces. For each microsphere, the interface is either fully bonded or fully debonded.
2. Partially debonded model (Figure 1b): Each microsphere has an interface which consists of two distinctly divided regions of bonded and debonded states. When the debonded region is smaller than the bonded region, there is a cap-type gap in one side of the interface. The cap-type connection exists in the opposite situation.
3. Discontinuously bonded model (Figure 1c): The interface is discontinuously bonded meaning the microsphere and matrix are sparsely interconnected at the interface.

The microstructure of each of the above cases was described by the representative unit cell approach for the FEM modeling. For the mixed model, the previously developed multi-particle unit cell approach was used [18,19,20]. A random sequential adsorption algorithm [27] was used to generate a random distribution of microspheres. Moreover, it was assumed that the unit cell is representative of the periodically arrayed whole microstructure. Therefore, a microsphere which penetrated the cell boundary was cut by the boundary and reappeared through the opposite side of the boundary.

**Figure 2:**
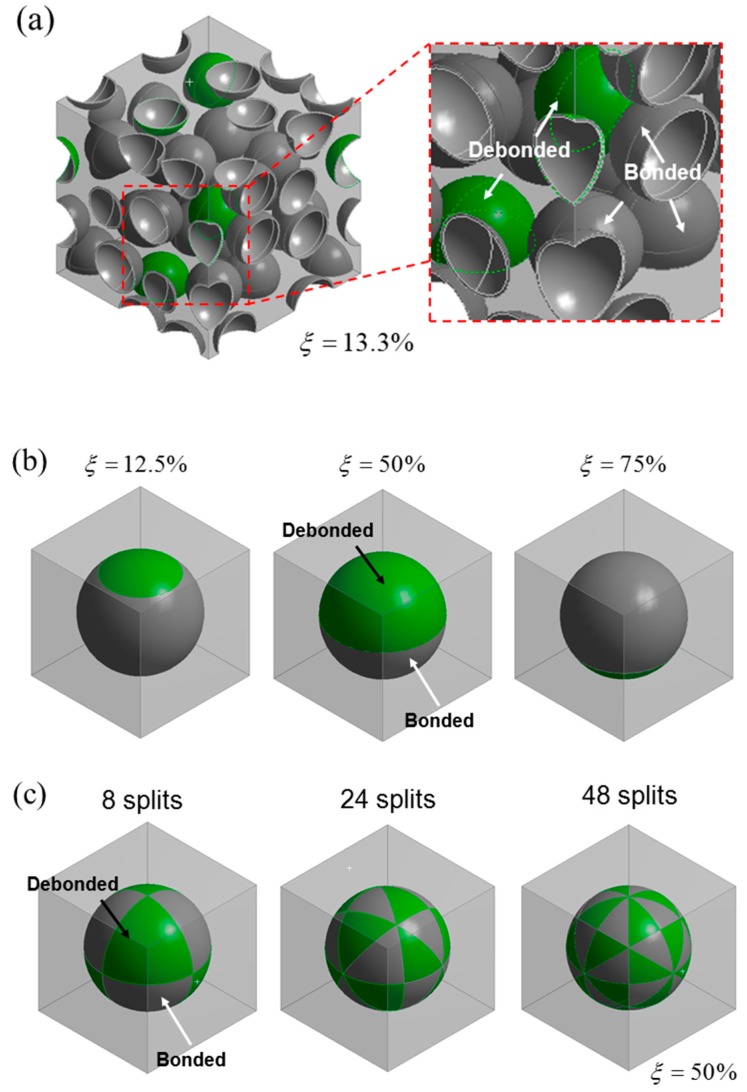
(**a**) Model geometry for the mixed model, with 30 randomly distributed microspheres, showing the debonded geometry for a debonding fraction of 13.3% (four microspheres out or a total of 30 microspheres are debonded); (**b**) Representative model geometries of the partially debonded model for ξ = 12.5%, 50% and 75%; (**c**) Model geometries for the discontinuously bonded models.

The geometry of the mixed model is shown in Figure 2a. The model consists of cubic unit cell containing 30 microspheres, with a microsphere volume fraction, f, of 30%. The mesh was made finer close to the interface in order to capture precisely the interaction between the microsphere and the matrix.

In this study, a debonding fraction, ξ, is defined by the area of the debonded interface divided by the total interface area. In the mixed model, the debonded interfaces were set by choosing the microspheres one by one randomly, and applying the debonded condition to their interfaces. Thus, in this case the model with higher ξ means that more microspheres have debonded interfaces.

The microstructures for the partially debonded and discontinuously bonded models were idealized by the single particle unit cell approach. For both of the models, f = 30% was used. The geometries of the models are shown in Figure 2b,c. A microsphere embedded cubic cell was used for both the models. The ξ in the partially debonded model was varied by adjusting the distance between the top of the microsphere and the bonded-debonded interface line in the model, as shown in Figure 2b. In the discontinuously bonded model, a sparsely interconnected interface was idealized by three different geometries where the interfaces were divided into eight, 24, and 48 splits having equal face areas. The divisions of the interfaces were done by 90°, 60°, and 45° inclined planes for the eight, 24, and 48 splits, respectively, as shown in Figure 2c. The debonded interface was modeled by applying the debonded condition to only half of the total faces of the splits, so that the interconnected areas had chess board-like structures and the ξ in this model was fixed to 50% for all cases. More details of the finite element mesh used in the study including mesh density and the mesh dependency analysis can be found in the supplementary document.

**Figure 3:**
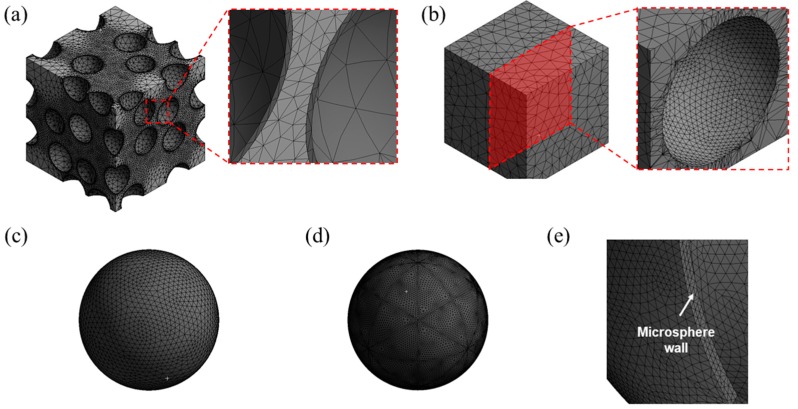
Finite element meshes for (**a**) the mixed model; (**b**) matrix for the partially debonded and discontinuously bonded models; (**c**) microsphere of the partially debonded model; (**d**) microsphere of the discontinuously bonded model; and (**e**) cross-section image of meshes used for the microsphere wall in the partially debonded model.

The models used in the study were discretized by finite elements with an automatic meshing algorithm provided by a commercial finite element code Ansys (version 14.5), as shown in Figure 3. Higher-order, 10-node tetrahedral elements with four integration points (SOLID187, provided by the Ansys) were used to mesh the models. The meshes were made finer in the matrix near the interface to the microsphere in order to accurately capture the contact behavior between the matrix and the microspheres. For the mixed model, the meshes were made by first dividing the interfaces with a mesh density of 1/20 of the diameter of the microsphere. Then, volumetric meshes were made from the surface meshes using the automatic meshing algorithm. The mixed model used in the study had 1,074,541 nodes and 635,343 meshes. As shown in Figure 3a, each microsphere had only two finite elements in the thickness direction. This was because of the geometrical complexity of the model and the limited mesh density that can be adopted for the calculations. The use of higher-order elements was thus necessary to capture the bending of thin microsphere walls. The validity of the finite element meshes used for the mixed model was checked by comparing with the models in which the microspheres were meshed with brick and shell type elements. The results are described in the supplementary document.

The geometries of the partially debonded and discontinuously bonded models were simpler than the mixed model. Hence, finer meshes were able to be adopted for the calculations, as shown in Figure 3b–e. The meshes of the microspheres were made finer than the mixed model, in order to avoid any undesirable stress concentration near the edge of the bond-debond interface. For each model, approximately 45,000 nodes and 22,000 meshes were used.

One important factor for determining elastic properties, using the representative unit cell approach, is choosing the appropriate type of boundary conditions. In the literature, the kinematic uniform, static uniform, orthogonal mixed, and periodic boundary conditions have been frequently used [28,29,30]. As indicated by Huet [28], the orthogonal mixed boundary condition provides better estimates, along with the periodic boundary condition, than the other two types of boundary conditions. Another advantage of using the orthogonal mixed boundary condition is that it can straightforwardly describe the uniaxial tension and compression behavior with the Poisson effect, using the FEM, without any special treatment. Whereas when using the periodic boundary condition, the Poisson effect, which involves tension or contraction in transverse direction to the loading in uniaxial deformation, is not directly demonstrated and requires special consideration. Therefore, the orthogonal mixed boundary condition was used throughout this study. The elastic properties of each model were estimated using the FEM by applying a 1% tensile and compressive uniaxial strain to the model. Due to the orthogonal mixed boundary condition and the symmetry of the applied load, all of the six outside planes of the cubic unit cells remain on plane after deformation.

The calculations were performed with version 14.5 of the finite element code Ansys, by means of a static structural analysis of the uniaxial deformation. The surface-to-surface interaction between the microsphere and the matrix in the debonded area was modeled by the Coulomb frictional model. In the debonded area, the load was only transferred when contact occurred between the microsphere and the matrix. In the simulations, the contact status was monitored by checking the relative positions of the nodes of the microsphere on the debonded interface to the interface surface of the matrix, and contact occurred when the node penetrated the matrix surface. An augmented Lagrangian contact algorithm was used in both normal and tangential directions. A hard contact condition was defined in the direction normal to the interface plane, meaning the load normal to the interface was perfectly transferred by the contact forces when the two surfaces were in contact. A normal contact stiffness factor of one was used, which is the penalty stiffness relative to the matrix stiffness that acts in the normal direction on the matrix surface, and enforces the displacement compatibility by limiting the penetration of the matrix phase. For the Coulomb friction model, a negligibly small friction coefficient of 0.001 was used for better convergence to restrict the rotational movement of the microsphere without provoking any pronounced effect due to friction.

The glass microsphere-filled polymer foam, with an elastic modulus of 3.2 and 72 GPa for the matrix and the microsphere, respectively, were used for the test cases throughout the study. The Poisson’s ratios for the matrix and the microsphere were 0.33 and 0.22, respectively. The radius ratio, which is the ratio of inner to outer radius of the microsphere, η, of 0.95 was used for the calculations.

## 3. Numerical Results

### 3.1. Mixed Model

**Figure 4:**
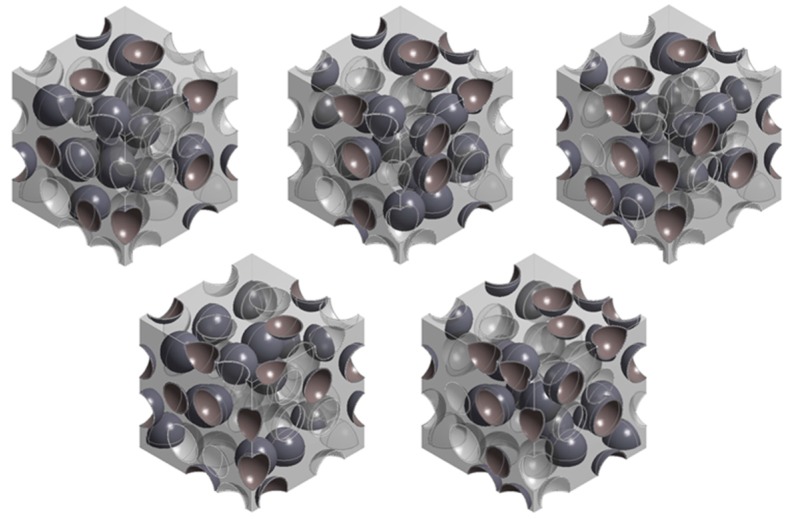
Five different representative elementary volumes (RVEs) for the mixed model, having the same ξ = 50% with differently selected debonded microspheres. In the figure, bonded microspheres are shown in dark grey and debonded microspheres are transparent.

In order to ensure the statistical homogeneity of the model associated by randomly distributed debonded microsphere, five different representative volume elements (RVEs), having the same ξ of 50% but with different arrangements of debonded microspheres, were built (Figure 4). The ξ = 50% was chosen because the randomness occurred due to the random arrangement of the debonded microspheres was expected to be the highest in this condition. Table 1 shows the compressive and tensile moduli obtained by the five different RVEs shown in Figure 4. The standard deviations of the moduli were ~3.7 MPa in compression and ~7.0 MPa in tension, which are negligibly small compared to the obtained moduli, accounting for less than 0.3% of the averaged modulus in tension and even less in compression. This indicates that the RVE consisting of 30 microspheres is large enough to statistically represent the behavior of the syntactic foam with randomly arranged debonded microspheres.

**Figure 5:**
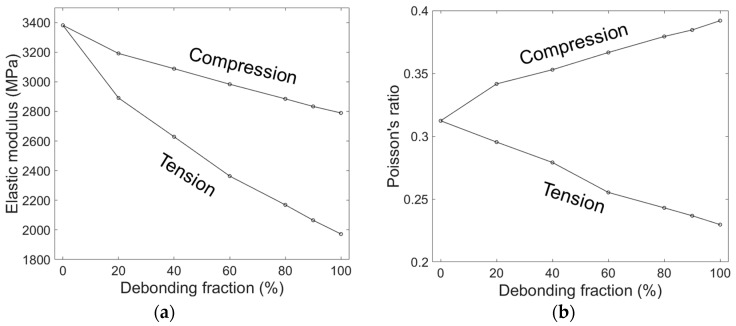
(**a**) Elastic modulus and (**b**) Poisson’s ratio obtained by the mixed model for a microsphere volume fraction of 30%, as a function of debonding fraction in tension and compression.

**Figure 6:**
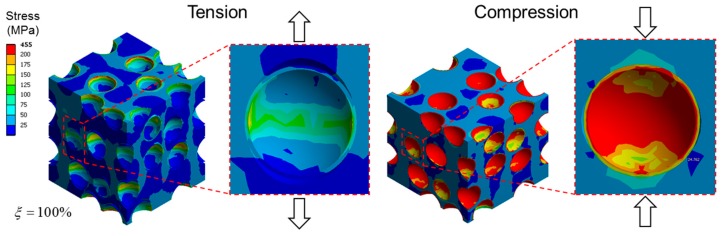
Equivalent stress fields at 1% tensile and compressive loads for the mixed model, with ξ = 100% which is complete debonding for all microspheres. Strains are magnified by a factor of 10 for better visualization.

Figure 5 shows the evolution of the elastic modulus and the Poisson’s ratio obtained by the mixed model with increasing ξ in tension and in compression. Both the compressive and tensile moduli decreased with increasing ξ. The tensile modulus decreased more sharply with ξ than the compressive modulus, which is believed to be due to a lower load transfer capability in tension than in compression associated with debonded interfaces. While under tension, the applied load caused a pair of cap-type gaps at the debonded interface, and the load transfer from the matrix to the microsphere occurred only by the compressive forces perpendicular to the loading, associated with the Poisson effect of the matrix. Conversely, in compression, the applied load can directly transfer from the matrix to the microsphere by compressing the microsphere along the loading direction, and the reduction of the overall stiffness occurs only by the effect of lesser CONFINEMENT in the direction perpendicular to the loading. This effect is clearly indicated in Figure 6, where the deformed geometries, and the equivalent stress fields in tension and compression, are compared. In the figure, the cap-type interfacial gaps between the microsphere and the matrix are clearly seen in tension, while in compression only thin cracks perpendicular to the loading were observed. It can be also seen that the stress in the microsphere is much higher in compression than in tension, indicating that more load is transferred to the microsphere.

The Poisson’s ratio shown in Figure 5b was calculated by measuring two independent apparent displacements of the model side walls perpendicular to the loading axis, and taking the average of the two measured values. The variation between the two obtained values for the Poisson’s ratio was very small - less than 0.01 - for every case indicating that the multi-particle unit cell used here has a sufficiently large number of microspheres to have nearly isotropic behavior. As shown in Figure 5b, the Poisson’s ratio under tension decreased with ξ, while the Poisson’s ratio under compression increased. This can be explained by the formation of the interfacial gaps associated with the deformations, which expands the overall volume of the model by introducing gaps into it. The volumetric expansion during deformation causes a reduction in the Poisson’s ratio in tension, which is the opposite in compression.

### 3.2. Partially Debonded Model

As shown in Figure 2b, the partially debonded model has a microsphere in which the debonding occurs in one direction only. Considering its axisymmetric geometry, the model can be considered as a transversely isotropic material with five independent elastic constants: elastic modulus and Poisson’s ratio in the isotropic plane ($E_{t}$ and $\nu_{tt}$); elastic modulus, Poisson’s ratio and shear modulus associated with the out-of-plane direction ($E_{l}$, $\nu_{tl}$ and $G_{tl}$), where the subscript t and l refer to transverse and parallel directions to the normal direction of the plane of axisymmetry.

**Figure 7:**
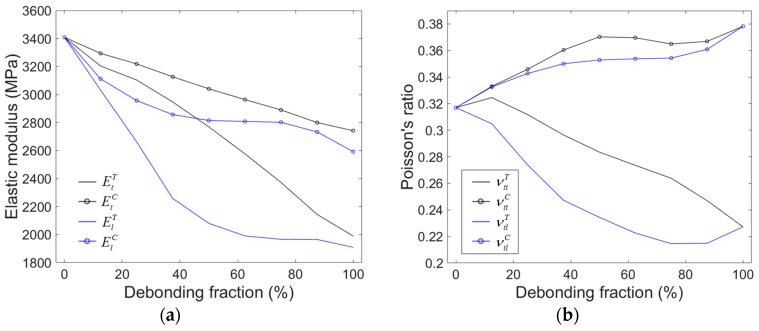
(**a**) Elastic moduli and (**b**) Poisson’s ratios obtained by the partially debonded model for a microsphere volume fraction of 30%, as a function of the debonding fraction. Black and blue lines represent directions in the isotropic plane and out-of-plane, respectively. Lines with and without circular marks indicate compressive and tensile elastic components, respectively.

Figure 7 shows the elastic moduli and the Poisson’s ratios for the models as a function of ξ. For the symbols used in the figure, the superscript *T* and *C* indicate the moduli obtained from tension and compression, respectively. The elastic properties in the isotropic plane, i.e., $E_{t}$ and $\nu_{tt}$, were obtained by applying loads to the models in the direction parallel to the debonded interface. The out-of-plane properties, i.e., $E_{l}$ and $\nu_{tl}$, were obtained by applying loads in the direction normal to the debonded interface.

As can be seen in the figures, deviations between $E_{t}$ and $E_{l}$ are more significant under tension than under compression. Similar behavior can be also found in the Poisson’s ratios. Both the tensile and compressive moduli decreased with increasing ξ. The Poisson’s ratio decreased in tension and increased in compression, which is the same trend seen in the results from the mixed model.

The single particle unit cell assumes that all the debonding gaps are aligned perfectly along one direction in the syntactic foam. For the foam having randomly oriented debonding gaps, the moduli can be estimated by taking the orientational average of the mechanical strain concentration tensor, as shown in prior studies [31,32]. The non-zero components of the compliance tensor for the single particle unit cell, $S_{ijkl}$ (i,j,k,l = 1,2,3) in the coordinates (x,y,z) are given by:

(1) $$\begin{array}{l} S_{1111}=\frac{1}{E_{l}},\;S_{1122}=S_{1133}=-\frac{\nu_{tl}}{E_{l}},\;S_{2222}=S_{3333}=\frac{1}{E_{t}},\;S_{2233}=-\frac{\nu_{tt}}{E_{t}},\;\\ 2\cdot S_{2323}=S_{2222}-S_{2233}=\frac{2\left(1+\nu_{tt}\right)}{E_{t}},\;S_{1212}=S_{1331}=\frac{2}{G_{tl}} \end{array}\}$$

The compliance components satisfy the following symmetry relations

(2) $$S_{ijkl}=S_{klij}=S_{jikl}=S_{ijlk}$$

**Figure 8:**
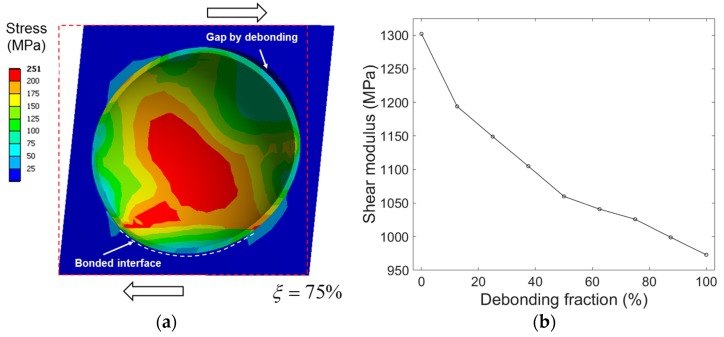
(**a**) Cross-section view of the equivalent stress field of the partially debonded model subjected to 1% shear loading (ξ = 75 %). Strain is 10 times larger than actual for easier visualization. (**b**) Evolution of shear modulus $G_{tl}$ with increasing ξ.

**Figure 9:**
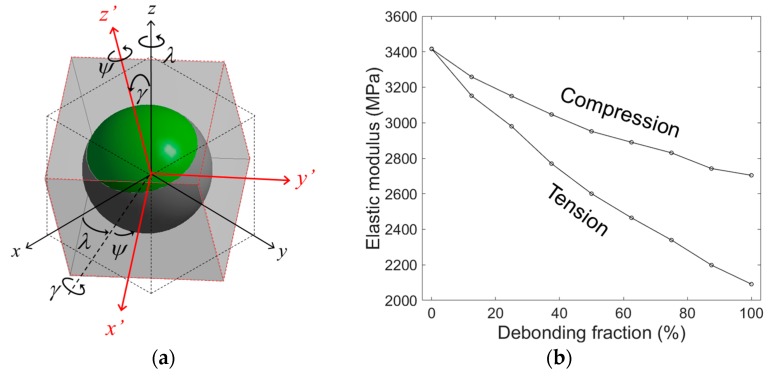
(**a**) Schematic of rotation of coordinates and (**b**) elastic moduli of the partially debonded model with random orientation of debonding direction.

For the calculations, the independent shear modulus, $G_{tl}$, was estimated by applying a pure shear strain of 1% to the models. As similar to the uniaxial loading cases, all the outer faces of the models were constrained to have flat surfaces during shear loading. Figure 8a shows the deformed shape and the equivalent stress distribution of the model under shear loading. The resulting evolution of the shear moduli with increasing ξ is shown in Figure 8b. The effective elastic compliance tensor for the randomly oriented partial debonding, denoted by superscript *, is given in prior studies [31,32]:

(3) Sijkl*=12π∫−ππ∫0π∫0π2aipajqakralsSpqrssin(γ) dλdγdψ

where λ, γ and ψ are the angles indicating the transformation from the unit cell local coordinates (x,y,z) to the global coordinates ($x^{\prime },y^{\prime },z^{\prime }$) (Figure 9a). $a_{ij}$ is the direction cosine for the transformation given by

(4) $$\begin{array}{l} a_{11}=\cos\left(\lambda \right)\cos\left(\psi \right)-\sin\left(\lambda \right)\cos\left(\gamma \right)\sin\left(\psi \right),\\ a_{12}=\sin\left(\lambda \right)\cos\left(\psi \right)-\cos\left(\lambda \right)\cos\left(\gamma \right)\sin\left(\psi \right),\;\\ a_{13}=\sin\left(\psi \right)\sin\left(\gamma \right),\\ a_{21}=-\cos\left(\lambda \right)\sin\left(\psi \right)-\sin\left(\lambda \right)\cos\left(\gamma \right)\cos\left(\psi \right),\\ a_{22}=-\sin\left(\lambda \right)\sin\left(\psi \right)+\cos\left(\lambda \right)\cos\left(\gamma \right)\cos\left(\psi \right),\\ a_{23}=\sin\left(\gamma \right)\cos\left(\psi \right),\\ a_{31}=\sin\left(\lambda \right)\sin\left(\gamma \right),\\ a_{32}=-\cos\left(\lambda \right)\sin\left(\gamma \right),a_{33}=\cos\left(\gamma \right) \end{array}\}$$

The resulting elastic compliance tensor is isotropic, which is the averaged value for all the directions in three dimensions. The orientation-averaged elastic modulus for the randomly oriented partial debonding can be obtained from the resulting compliance tensor as follows:

(5) $$E^{*}=\frac{1}{S_{1111}^{*}}=\frac{1}{S_{2222}^{*}}=\frac{1}{S_{3333}^{*}}$$

The elastic moduli calculated by Equation (5) for models having a different ξ are plotted in Figure 9b, for tension and compression.

### 3.3. Discontinuously Bonded Model

The compressive and tensile elastic moduli of the discontinuously bonded model were obtained with a different number of splits, and are listed in Table 2. The behavior of the model was closely isotropic because the debonded areas were quasi-uniformly distributed in all directions. For different model geometries, the compressive modulus of the model remained unchanged at around 2920 MPa, whereas the tensile modulus increased notably from 2767.4 to 2934.9 when the number of splits increased from 8 to 48. The Poisson’s ratios of the discontinuously bonded model was found to be nearly constant at 0.31 ± 0.05.

## 4. Discussion

**Figure 10:**
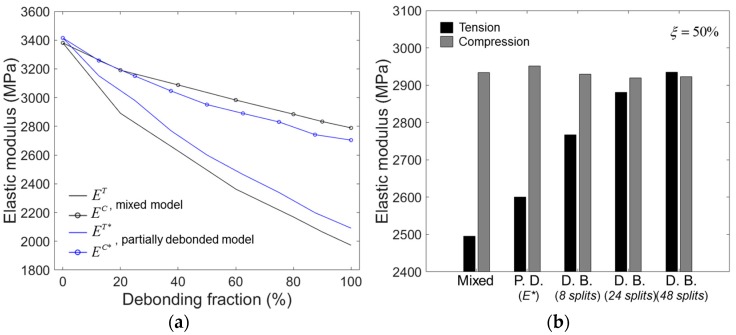
(**a**) Elastic moduli as a function of ξ for the mixed and partially debonded models. (**b**) Compressive and tensile moduli for ξ = 50%, obtained by different models. The abbreviations used are: P.D. (partially debonded) and D.B. (discontinuously bonded).

Figure 10 compares the elastic moduli obtained by the models with different debonding geometries. In Figure 10a, the elastic moduli obtained by the mixed and the partially debonded models are plotted as a function of ξ. For the partially debonded model, the elastic moduli for the randomly oriented partial debonding ($E^{*}$) were used for comparison. The two models showed almost identical elastic moduli for ξ = 0% (perfect bonding), slightly deviating from each other for higher ξ. The compressive moduli obtained by the mixed model was slightly higher than those obtained by the partially debonded model, while the opposite trend was observed for the tensile moduli. However, the deviations in the moduli between the two models were not significant and the two models were fairly well correlated.

Figure 10b shows the compressive and tensile moduli obtained by all the models tested for ξ = 50%. It is seen that the compressive modulus is not altered much by changing the debonding geometry, indicating that it almost solely depends on the debonding fraction. In contrast, the tensile modulus strongly depends on the debonding geometry, as shown in the figure. For the fixed ξ = 50%, the tensile modulus was highest in the mixed model, followed by the partially debonded model, then the discontinuously bonded model with eight, 24, and 48 splits. Given these results, it seems that the tensile modulus relies largely on the relative size of the debonding gap in the model. In the mixed model, each debonding gap’s size corresponded to the full interface area between the microsphere and the matrix. For the other models, the individual debonding gap’s size, in relation to the full interface area, was smaller in the inverse order of their tensile moduli, i.e., 1/2, 1/8, 1/24, and 1/48 for the partially debonded and discontinuously bonded models with eight, 24, and 48 splits, respectively.

**Figure 11:**
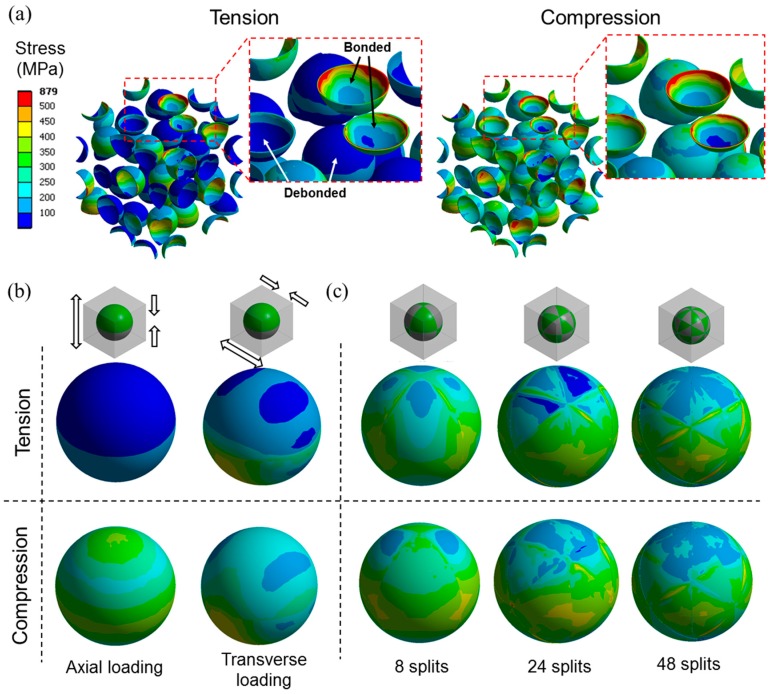
Equivalent stress distributions in microspheres for models with ξ = 50%. (**a**) Mixed model; (**b**) partially debonded model; and (**c**) discontinuously bonded model.

Figure 11 shows the stress distributions in the microspheres for the same models used in Figure 10b. In the mixed model, the microspheres with the debonded interfaces clearly showed less stress compared to the ones with the bonded interfaces under tensile load (Figure 11a). This is because the applied load is more efficiently transferred to the microspheres having bonded interfaces than those that were debonded. The stress in the debonded microspheres was much less under tension than compression, indicating that the low tensile modulus of the mixed model under compression can be attributed to the poor load bearing efficiency by the debonded microspheres. This effect is more clearly seen in Figure 11b, where the stress distribution of the half debonded microspheres in the partially debonded model, in two different loading directions, are presented. With the tensile load in the direction normal to the axisymmetric plane of the model, also known as axial loading, the model exhibited similar behavior to the mixed model, i.e., the stress near the debonded interface was significantly lower under tension than compression due to the poor load bearing efficiency under tension. When the load was applied in the direction perpendicular to the axisymmetric plane, also known as transverse loading, more load was transferred through the debonded interface. The load transfer by the debonded interface becomes more effective when the debonded area is more finely divided, as shown in Figure 11c in the results of the discontinuously bonded model.

The results presented in Figure 10 and Figure 11 indicate that the load transfer with tensile loading through the debonded interface becomes more efficient with finer debonding gaps, for the same ξ. When the debonding gap is fine enough, the load transfer effect through the debonded interfaces is comparable for tension and compression, as shown by the discontinuously bonded model with the 48 split in Figure 11c. In this case, the resulting tensile modulus is therefore comparable to that for compression. The finer the debonding gaps, the smaller the gap under tensile load which makes more interfacial area remain in contact during deformation along with more efficient load transfer. This effect may not be dominant under compression since there is no gap formation in the loading direction. This explains why the compressive moduli are not much influenced by the debonding geometry, as shown in Figure 10b.

When predicting the elastic response of syntactic foam using a simple elastic model with a perfectly bonded interface, it overestimates the experimentally observed elastic moduli of the foam in most cases, as shown in [3,17,18,19,20]. Several reasons have indicated as possible factors leading to this overestimation, such as matrix defectiveness [19] and weak interfacial phase formation between the matrix and microsphere [22,23]. In considering the numerical results presented here, the partial interfacial debonding occurring during the processing, or during deformation, at low stress could also be considered as a possible cause for the underestimation. The reduction in elastic modulus by interfacial debonding can be as large as ~20% in the case of compressive loading, and up to 40% in with tension, for the considered syntactic foam as shown in Figure 10.

It was not possible to perform a direct comparison between the numerical results and the experiments for the purpose of verification, since experimental data for the detailed debonding geometry during loading is not available in the literature. However, the model used here intuitively considers the influence of debonding, which is helpful for gaining a better understanding of the effect of interfacial flaws on the performance of syntactic foam. The models can be easily adopted for syntactic foams having different microstructural parameters, such as for different microsphere wall thicknesses. As an example, a comparison between the mixed model and the analytical model were made and can be found in the supplementary document.

Notably, some experimental observations can only be explained by considering the partial debonding effect. For instance, Tagliavia et al. [3] investigated the effect of moisture absorption on the flexural properties of syntactic foams. They observed a 5–25% reduction in the flexural modulus due to a small amount of moisture absorption of less than 3 wt.%, for their syntactic foams with f = 30%. The moisture absorption is known to weaken the adsorption bond between a filler and matrix in a polymeric composite, as it forces itself between the filler and matrix and lowers the interfacial fracture energy, making it easier to debond [32]. The deterioration of the elastic modulus by this mechanism can be better characterized and quantified by analyzing the tensile and/or compressive modulus before and after the moisture absorption using the model used here. For another example, Rousseau et al. [33] experimentally determined the compressive moduli of epoxy based syntactic foams with various volume fractions of the glass microsphere up to 30%, using three different types of glass microspheres. In their results, all three types of foams showed linearly varied compressive moduli, in relation to the volume fraction, up to 20%, but this was followed by a pronounced drop in the compressive moduli when increasing the volume fraction from 20 to 30%. Since the materials and processing parameters used to fabricate the samples with 30% were the same as the others, the drop of the compressive moduli is not likely due to the weak interfacial phase formation. The void fraction due to the air entrapment during the fabrication process was at a maximum with only 2.2 % for every specimen. Thus the behavior cannot be solely explained by the matrix defectiveness but can easily interpreted when considering interfacial debonding. Notably, the syntactic foam with η = 0.95 [33] showed the compressive modulus of ~2800 MPa for f = 30%, which corresponds to the compressive modulus estimated by the partially debonded model with ξ = 70 %.

## 5. Conclusions

In this paper, a detailed numerical study on the elastic properties of the syntactic foam with a debonded interface has been presented. Three types of FEM models were used to predict the overall elastic behavior of partially debonded hollow microsphere reinforced syntactic foam. The multi particle unit cell was applied to model the foam, consisting of fully-bonded and debonded microspheres, while the models having an interfacial gap on each microsphere, or sparsely interconnected microspheres, were investigated through the single particle unit cell approach. All the models showed a reduction of the elastic modulus when increasing the ratio of debonded area to the total interfacial area between the microsphere and the matrix. The tensile modulus was considerably influenced the debonding geometry, while the compressive modulus was almost unaffected by it. For the fixed ratio of debonded area to the total interfacial area, a higher tensile modulus was obtained for the smaller interfacial gap size in relation to the size of the microsphere. When the interfacial gap was much smaller than the size of the microsphere, there was no disparity between the tensile and compressive moduli. Although the models used in this paper have not yet been validated due to the lack of experimental data for the detailed debonding geometry of the syntactic foam during loading, some experimental observations on the elastic modulus being less than the theoretical value could be well explained by the numerical results from the models.

## Figures and Tables

**Table 1:** Compressive and tensile elastic moduli obtained by the mixed model for ξ = 50% with five different RVEs consisting of randomly selected debonded microspheres.

	Model No.1	Model No.2	Model No.3	Model No.4	Model No.5	Average
Compressive modulus (MPa)	3035.8	3027.8	3033.5	3033.5	3039.2	3033.9 ± 3.7
Tensile modulus (MPa)	2496.1	2504.8	2510.3	2500.4	2515.7	2505.5 ± 7.0

**Table 2:** Compressive and tensile elastic moduli obtained by the discontinuously bonded model.

Model Type	8 Splits	24 Splits	48 Splits
Compressive modulus ($E^{c}$, MPa)	2929.4	2919.4	2922.5
Tensile modulus ($E^{t}$, MPa)	2767.4	2881.2	2934.9

## Supplementary Materials

The following are available online at www.mdpi.com/1996-1944/10/8/911/s1, Figure S1: Models with different finite element meshes on microspheres, Figure S2: Comparison of tensile moduli obtained by mixed model for ζ = 0% (fully bonded) with analytical models with 30% microspheres without void, Figure S3: Comparison of tensile moduli obtained by mixed model for ζ = 33.3%. The mixed model had microsphere volume fraction of 30% in total (bonded microspheres by 20% and debonded microspheres by 10% in volume fraction), Figure S4: Comparison of tensile moduli obtained by mixed model for ζ = 66.6%, Table S1: Tensile elastic moduli obtained by mixed models with different finite element meshes in microspheres.
