# Supplementary material for: Influence of Partially Debonded Interface on Elasticity of Syntactic Foam: A Numerical Study
Source: Materials (Basel). 2017 Aug 8;10(8):911. doi: 10.3390/ma10080911 (PMC5578277; doi:10.3390/ma10080911)
Supplement: Supplementary file 1 [file materials-10-00911-s001.pdf]

Supplementary Materials: Influence of Partially Debonded Interface on Elasticity of Syntactic Foam: A Numerical Study

Yi Je Cho, Youngjeong Kang, Young Cheol Lee, Yongho Park and Wookjin Lee

1. Mesh validation for mixed model

For the mixed model, the meshes in the microspheres were made by first dividing the interfaces with a mesh density of 1/20 of the diameter of the microsphere. Then the volumetric meshes were made from the surface meshes by the automatic meshing algorithm provided by the Ansys. Since the wall thickness of the microsphere is thinner than the surface mesh density, the microspheres were meshed to have only two finite elements in the thickness direction (shown in Fig.3a in the paper). Since each tetrahedral element had 4 integration points near its corners, there is only two or three integration points through the thickness direction of the microspheres in the mixed model.

The validity of the finite elements used in the mixed model was checked by comparing with models having different types of finite elements meshes in the microspheres. Four different models were used for the comparison. Among them, three models used brick-type elements for the microspheres with 2, 4 and 6 layers in the thickness direction. The microspheres were meshed by 20-node brick elements (SOLID186, Ansys). In an additional model, the microspheres were discretized by two layers of 8-node solid-type multilayer elements (SOLSH190, Ansys). The finite element meshes of the different models are shown in Fig.S1. The elastic modulus of the models were obtained by applying 1% uniaxial tensile loads. The boundary conditions were the same as described in the paper. The debonding fraction was fixed for the all calculation by ζ = 0% (fully bonded).

Table.S1 shows the elastic moduli obtained by the different models and their deviations from the mixed model used in the paper (tetrahedrons). The resulted moduli are almost identical in all the models tested. The deviations are less than 0.3% which is negligible for the purpose of estimating the elastic modulus, indicating that the tetrahedron elements used in the mixed model in the paper can appropriately describe the deformation behavior of the microspheres.

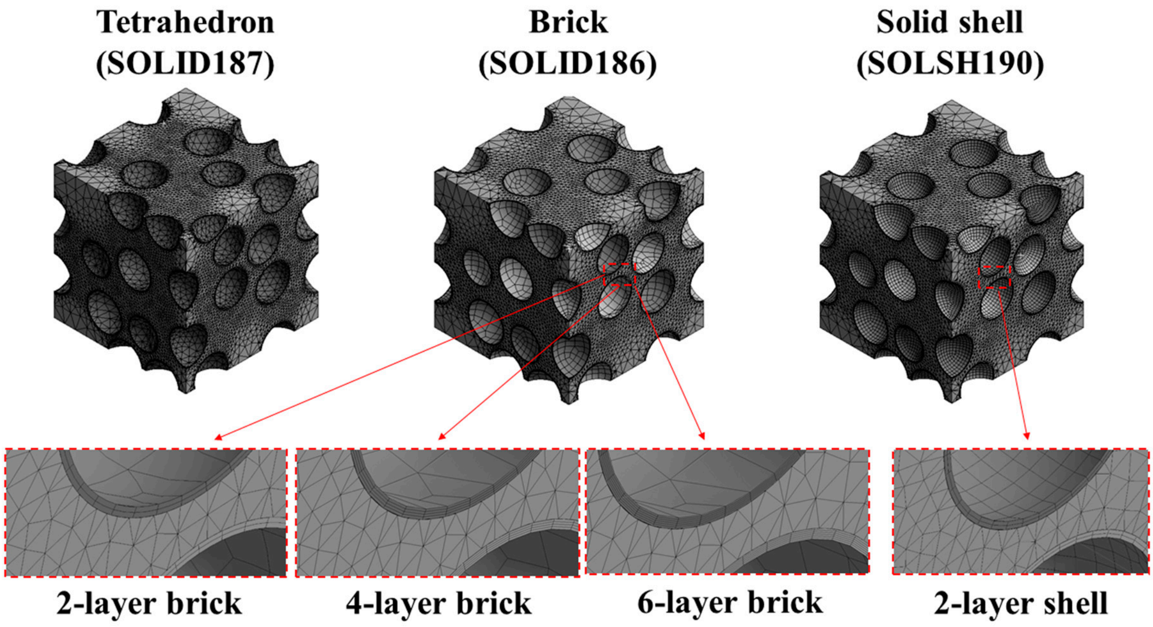

Figure S1. Models with different finite element meshes on microspheres. The model with tetrahedrons has the same meshes used for the mixed model in the paper (upper left side).

Table S1. Tensile elastic moduli obtained by mixed models with different finite element meshes in microspheres.

	Tetrahedron (used in the study)	Brick with 2-layer	Brick with 4-layer	Brick with 6-layer	Shell with 2-layer
Modulus (MPa)	3386.7	3381.4	3381.2	3380.3	3377.4
Deviation from tetrahedron (%)	-	0.16	0.16	0.19	0.27

2. Comparison of the result with analytical model

The model used in the paper is compared with existing analytical model in the literature. The analytical model used for the comparison was previously developed by Aureli et al. [S1] for predicting elastic responses of syntactic foam consisted of both microspheres and spherical voids. The mixed model was used for the tensile elastic moduli as a function of debonding fraction, ζ . Three different radius ratios (η) of 0.93, 0.95 and 0.97 were considered.

In the mixed model used for the comparison, only the thickness of the microsphere wall was changed and all the other geometrical parameters were the same to the model used in the paper. The material parameters used for the calculations were selected as in [S1]: the elastic moduli of 3.52 and 60 GPa and the Poisson's ratios of 0.3 and 0.21, for the matrix and the microsphere, respectively.

Fig.S2 plots tensile moduli obtained by the mixed model with ζ = 0% (fully bonded), along with the prediction of moduli of void free syntactic foam with microsphere volume fraction of 30% [S1]. In the figure, the curve 'nominal microsphere' shows results obtained by analytical model using the nominal microsphere wall thickness provided by the manufacturer. The curve 'polydispersed' represents the analytical result with the polydisperse scheme with the experimentally determined wall thickness distribution. It can be seen that the moduli obtained by the mixed model agree fairly well with the two analytical models when assuming the fully bonded condition.

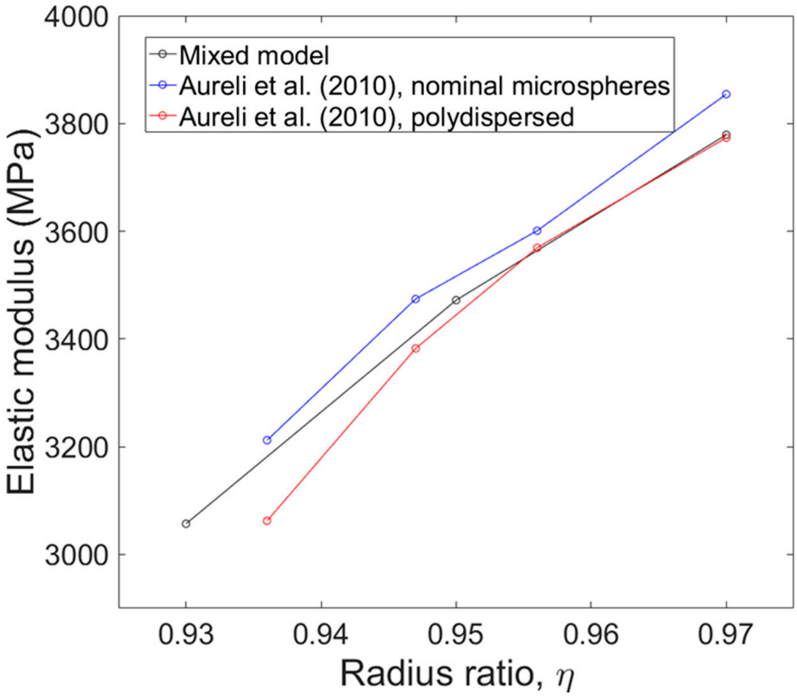

Figure S2. Comparison of tensile moduli obtained by mixed model for ζ = 0% (fully bonded) with analytical models with 30% microspheres without void in [S2].

Figs.S3 and 4 plots the tensile modulus obtained by the mixed models with debonding fractions of 33.3 and 66.6%, respectively. For comparisons, the analytical models for the syntactic foam containing both microsphere and voids are also plotted. In Fig.S3, the mixed model with debonding fractions of 33.3% was used. In other words, the mixed model used in Fig.S3 consisted of bonded microspheres by 20% in volume fraction and debonded microspheres by 10% in volume fraction, if the volume fractions of the bonded and debonded microspheres are considered separately. The analytical results shown in the figure were obtained by assuming that the syntactic foam has microspheres by 20% in volume fraction and voids by 10% in volume fraction. Therefore, the figures compare the models with the same (bonded) microspheres having the same volume fraction of the defects (debonded microspheres or voids). In Fig.S4, both the models had 10% of bonded microspheres but the mixed model had 20% of debonded microspheres while the analytical model consisted of 20% of voids.

In both Fig. S3 and S4, the analytical model showed the same trend to the results obtained by the mixed model. The moduli obtained by the mixed model were always higher than the analytical results and the deviation between the models becomes wider when the debonding fraction (or the void fraction in the analytical model) is increased. The reason for this deviation is obvious from Fig.3 of the paper, which showed that the microsphere can bear loads under tension by lateral contact even when the interface was completely debonded.

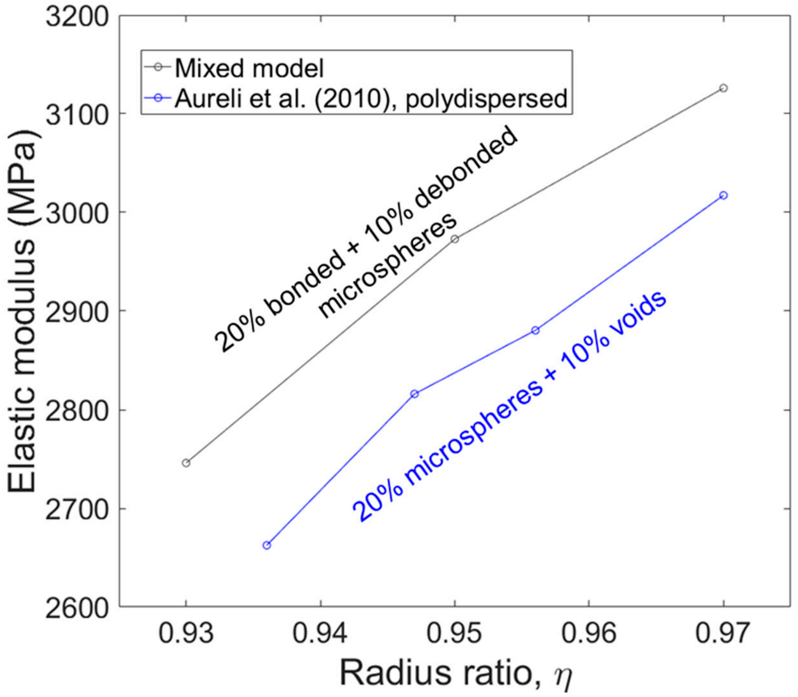

Figure S3. Comparison of tensile moduli obtained by mixed model for ζ = 33.3%. The mixed model had microsphere volume fraction of 30% in total (bonded microspheres by 20% and debonded microspheres by 10% in volume fraction). Analytical model assumed a syntactic foam having 20% of microspheres with 10% spherical voids [S2].

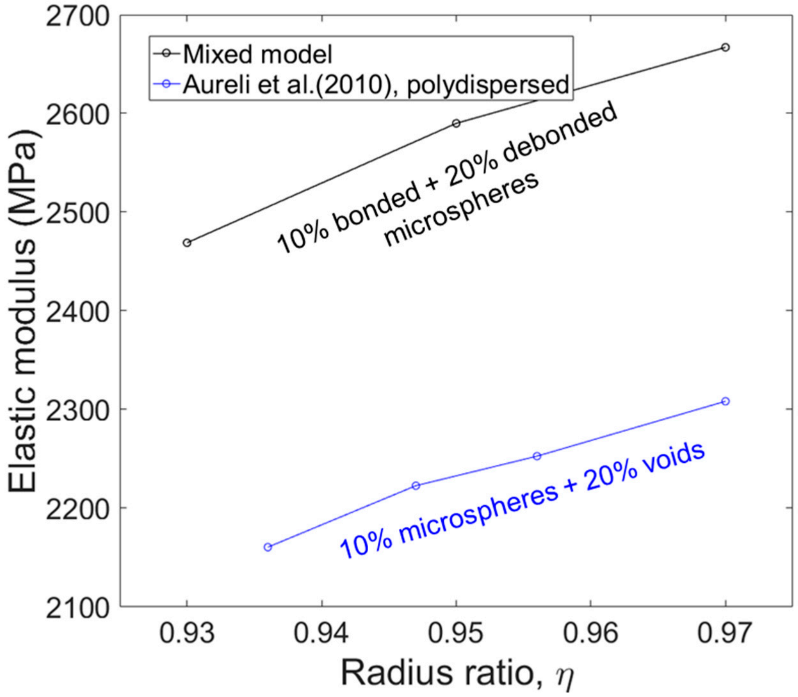

Figure S4. Comparison of tensile moduli obtained by mixed model for ζ = 66.6%. The mixed model had microsphere volume fraction of 30% in total (bonded microspheres by 10% and debonded microspheres by 20% in volume fraction). Analytical model assumed a syntactic foam having 10% of microspheres with 20% spherical voids [S2].

Reference

[S 1] Aureli M.; Porfiri M.; Gupta N. Effect of polydispersivity and porosity on the elastic properties of hollow particle filled composites. Mech. Mater. 2010, 42, 726-739.

© 2017 by the authors. Licensee MDPI, Basel, Switzerland. This article is an open access article distributed under the terms and conditions of the Creative Commons Attribution (CC BY) license (http://creativecommons.org/licenses/by/4.0/).
